# Alternative lengthening of telomeres: remodeling the telomere architecture

**DOI:** 10.3389/fonc.2013.00027

**Published:** 2013-02-20

**Authors:** Dimitri Conomos, Hilda A. Pickett, Roger R. Reddel

**Affiliations:** ^1^Cancer Research Unit, Children’s Medical Research InstituteWestmead, NSW, Australia; ^2^Sydney Medical School, University of SydneyNSW, Australia

**Keywords:** telomere, alternative lengthening of telomeres (ALT), chromatin, shelterin, DNA damage response (DDR), recombination, variant telomeric repeats, nuclear receptors

## Abstract

To escape from the normal limits on proliferative potential, cancer cells must employ a means to counteract the gradual telomere attrition that accompanies semi-conservative DNA replication. While the majority of human cancers do this by up-regulating telomerase enzyme activity, most of the remainder use a homologous recombination-mediated mechanism of telomere elongation known as alternative lengthening of telomeres (ALT). Many molecular details of the ALT pathway are unknown, and even less is known regarding the mechanisms by which this pathway is activated. Here, we review current findings about telomere structure in ALT cells, including DNA sequence, shelterin content, and heterochromatic state. We speculate that remodeling of the telomere architecture may contribute to the emergence and maintenance of the ALT phenotype.

## INTRODUCTION

The vast majority of human cancers utilize a telomere maintenance mechanism to compensate for the gradual telomere shortening that accompanies cellular proliferation, and thereby obtain an unlimited replicative capacity. This can be accomplished by up-regulation of the ribonucleoprotein telomerase which adds telomeric repeats onto the ends of linear chromosomes by reverse transcription of an RNA template molecule (Morin, 1989), or by the alternative lengthening of telomeres (ALT) pathway (Bryan et al., 1995). Immortalized human cell lines that utilize ALT exhibit numerous phenotypic characteristics that are consistent with the hypothesis that ALT involves homologous recombination (HR)-mediated DNA copying of a telomeric DNA template (Dunham et al., 2000). These characteristics include telomere length heterogeneity (Bryan et al., 1995, 1997), abundant extrachromosomal linear and circular telomeric DNA (Ogino et al., 1998; Tokutake et al., 1998; Cesare and Griffith, 2004; Wang et al., 2004; Henson et al., 2009; Nabetani and Ishikawa, 2009), an elevated frequency of telomere-sister chromatid exchange (T-SCE) events (Bechter et al., 2004; Londono-Vallejo et al., 2004), and the presence of a specific subclass of promyelocytic leukemia (PML) nuclear bodies, containing telomeric DNA, shelterin proteins, and HR factors including Mre11–Rad50–Nbs1 (MRN), termed ALT-associated PML bodies (APBs; Yeager et al., 1999). The template for synthesis of new telomeric DNA can be the telomere of a non-homologous chromosome (Dunham et al., 2000) or telomeric sequences elsewhere in the same telomere or the telomere of a sister chromatid (Muntoni et al., 2009), and we speculate that extrachromosomal telomeric DNA may also act as the copy template (Henson et al., 2002).

Telomere length maintenance is a characteristic of almost all cancers. Consequently, there is considerable interest in the use of telomere maintenance inhibitors as a broad-spectrum cancer therapy, and telomerase inhibitors have entered clinical trials (Ruden and Puri, 2012). However, telomerase inhibitors are unlikely to be effective for ALT tumors, and there is a possibility that telomerase-positive tumors will become resistant by activating ALT. This is supported by recent studies showing that telomerase extinction in mouse lymphomas results in emergence of ALT activity and other adaptive responses (Hu et al., 2012). Therefore successful therapeutic targeting of telomere maintenance in cancers will encompass the development of ALT inhibitors. This will be facilitated by insights into the molecular details of ALT and how this mechanism is activated. Furthermore, the possibility remains that ALT activity may also exist under normal physiological conditions, with evidence for the mechanism seen in the mouse zygote during the early cleavage steps post-fertilization (Liu et al., 2007), and most recently in the somatic cells of mice (Neumann et al., 2013). These data suggest that while some form of ALT activity may constitute a natural aspect of telomere biology, the mechanism may become dysregulated during cancer development. Here, we review aspects of normal telomere function and the current understanding of ALT, with particular emphasis upon the structural modifications that occur to the telomere during the activation and maintenance of ALT.

## TELOMERE CAPPING FUNCTION

Telomeres contain several kilobases of the repetitive sequence 5′-TTAGGG-3′, which are predominantly double-stranded, but terminate in a single-stranded 3′ overhang of the G-rich strand (Moyzis et al., 1988). This terminus can invade upstream duplex telomeric DNA and anneal to the complementary C-rich strand, resulting in the formation of a lariat structure known as a telomere loop (t-loop; Griffith et al., 1999). The t-loop is thought to protect the chromosome by sequestering the free end, thereby preventing it from being recognized as a break by the DNA damage response (DDR) proteins (de Lange, 2004). Telomeres may also form other higher order structures such as G-quadruplexes (Williamson, 1994).

The chromosome end is further protected by telomere-binding proteins, especially a six-subunit protein complex (consisting of the proteins TRF1, TRF2, TIN2, POT1, RAP1, and TPP1) known as shelterin (Palm and de Lange, 2008). The protection afforded to chromosome ends by the telomeric nucleoprotein complex is referred to as telomere capping. Telomeres become uncapped when they undergo excessive shortening, presumably because they are no longer able to form a protective higher order structure and/or bind sufficient shelterin and other telomere-associated proteins, or when telomere-binding proteins such as TRF2 or POT1 are depleted experimentally (Denchi and de Lange, 2007). Removal of the entire shelterin complex has demonstrated the complexity of the capping function, which inhibits processing by multiple pathways, including ataxia telangiectasia mutated (ATM), ATM and Rad3-related (ATR), non-homologous end-joining (NHEJ), HR, and resection (Sfeir and de Lange, 2012). Loss of capping function can be recognized by co-localization of the telomere with various markers of the DDR, such as phosphorylated histone H2AX (γ-H2AX) and tumor suppressor p53-binding protein 1 (TP53BP1), which is referred to as a telomere dysfunction-induced focus (TIF; Takai et al., 2003), or by NHEJ of chromosome ends.

## TELOMERE CAPPING FUNCTION IN ALT CELLS

Most ALT cells lack functional p53 and contain remarkably large numbers of TIFs (Cesare et al., 2009). Although ALT is associated with a relatively high level of genetic instability (Lovejoy et al., 2012), this is compatible with continued cell cycling, so it seems most likely that the TIFs represent an intermediate or transient state rather than representing fully uncapped telomeres. The TIFs in ALT cells can be partly suppressed by expression of exogenous TRF2 in a manner consistent with its ability to inhibit the function of the DDR protein, ATM. Many of these TIFs occur on telomeres that are not short, and are not suppressed by lengthening the shortest telomeres with exogenous telomerase (Cesare et al., 2009). These observations suggest that ALT cells contain telomeres with abnormal capping function, and raise the question whether these abnormalities are actually required for ALT activity.

Alternative lengthening of telomeres cells exhibit a very substantial increase in T-SCEs, although the rate of HR elsewhere in the genome is not increased compared to telomerase-positive cells (Bechter et al., 2003, 2004; Londono-Vallejo et al., 2004). Thus there appears to be a specific defect in the ability of the telomere cap in ALT cells to suppress telomeric HR, and given the proposed involvement of HR intermediates in ALT-mediated copying of telomeric template DNA, it is reasonable to speculate that this cap defect is essential for ALT. This defect does not result from mutations in KU70, TRF2, POT1, or RAP1 which are all wild-type and present at normal levels in ALT cells (Lovejoy et al., 2012), so other explanations must be sought.

TRF2 is of particular interest in the context of telomere capping in ALT because, in addition to its involvement in suppression of telomeric HR described previously, it has a role in the formation of t-loops and four-strand DNA junctions, and in the protection of these structures against enzymatic cleavage. This suggests that TRF2 may have a role in the regulation of telomeric recombination by both promoting t-loop formation and preventing resolution of telomeric recombination intermediates (Stansel et al., 2002; Fouche et al., 2006; Poulet et al., 2009). In addition, through its interaction with the helicases BLM and WRN, TRF2 is also involved in the unwinding of duplex telomeric DNA (Opresko et al., 2002) and potentially in the resolution of aberrant telomeric structures. The total level of TRF2 in ALT cells is not significantly different from other cells (Lovejoy et al., 2012), but the total quantity of telomeric DNA is significantly increased (Lau et al., 2012), and overexpression of TRF2 is able to suppress the formation of TIFs (Cesare et al., 2009). These observations suggest that the amount of TRF2 (and possibly of other shelterin components) relative to telomeric DNA is decreased in ALT cells, resulting in a partial functional deficiency that may contribute to the prevalence of intermediate-state TIFs in these cells, and an HR-permissive telomeric state.

## ABNORMAL DNA SEQUENCES IN ALT TELOMERES

The proximal regions of normal human telomeres are composed of variant repeats such as TGAGGG, TCAGGG, and TTGGGG (Allshire et al., 1989; Baird et al., 1995). These regions are hypervariable and reflect a high underlying mutation rate, predominantly involving base substitutions and simple intra-allelic expansions and contractions. Characterization of these events is possible due to linkage disequilibrium spanning these proximal regions, which has resulted in the evolution of a limited number of haploid lineages with related telomere sequence maps (Baird et al., 1995). Variant repeats are usually restricted to the proximal 2 kb of the telomere (Allshire et al., 1989); however, several studies have indicated that ALT telomeres may contain an abundance of abnormal DNA sequences. Firstly, the C-circle assay produced higher yields from some ALT cell lines following inclusion of deoxycytidine triphosphate (dCTP), indicating the presence of sequences other than TTAGGG in telomeric C-circles (Henson et al., 2009). In addition to variant repeats, telomeres of ALT cells are able to accommodate large amounts of non-telomeric sequences such as SV40 DNA (Fasching et al., 2005; Marciniak et al., 2005).

**FIGURE 1:**
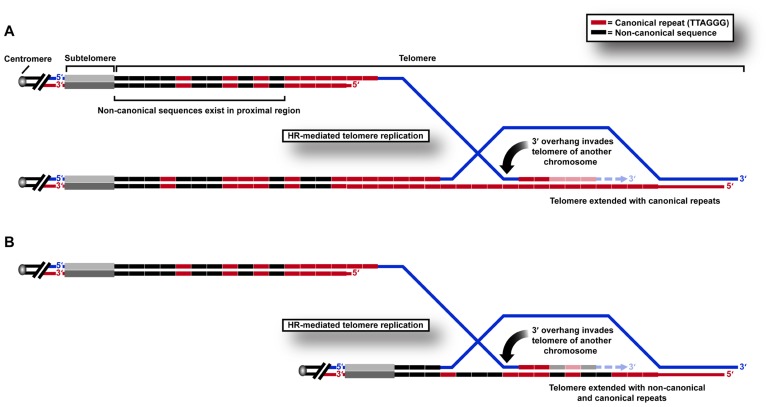
**HR-mediated telomeric replication in ALT cells**. Non-canonical sequences are present in the proximal 2 kb region of the telomere array in human cells. Telomeric replication in ALT cells can occur by HR-mediated inter-telomeric copying of **(A)** canonical TTAGGG repeats in the telomere repeat array, or **(B)** variant or non-canonical repeats present in the proximal regions of the telomere.

We recently used a sequencing approach to show directly that variant repeats are dispersed throughout ALT telomeres (Conomos et al., 2012). We propose that this results from the HR-mediated telomere replication that has previously been shown by telomere mapping experiments to occur in the variant repeat-dense proximal regions of the telomere (Varley et al., 2002). This can be predicted to cause a breakdown of linkage disequilibrium, and ultimately the spreading of variant sequences throughout the telomere (**Figure 1**) which may have profound implications for the structure and function of telomeric nucleoprotein.

One of the consequences of these changes may be to “lock in” a recombinogenic telomeric state. Telomere exchange events have been shown to occur at low frequency in normal telomere biology (Baird et al., 1995). We hypothesize that these may even more rarely involve the proximal telomere region, but that the frequency increases after genetic changes such as loss of p53 suppressor function. When variant repeats spread from the proximal telomere region in this way, they may destabilize the telomere in favor of recombination, resulting in the incorporation of more variant repeats and permitting further recombination, thereby creating a positive feedback loop that results in sustained ALT activity. This hypothesis is supported by telomere mapping analysis of clonal cell populations derived from an ALT cell line compared to pre-crisis cells, in which all clones contained a mutant telomere map, presumably as a result of a single early inter-telomeric recombination event during clonal expansion following crisis (Varley et al., 2002). The reason that a change in DNA content may result in increased telomeric recombinogenicity may lie in its effects on protein binding.

## ALTERED PROTEIN BINDING AT ALT TELOMERES

The shelterin complex binds specifically to the TTAGGG repeat sequence by means of the Myb domains in TRF1 and TRF2 which bind duplex telomeric repeats (Court et al., 2005; Hanaoka et al., 2005), and by sequence-specific binding of POT1 to single-stranded telomeric DNA (Loayza et al., 2004). Telomeres present a challenge to the DNA replication machinery, giving rise to replication-dependent defects, and they consequently resemble fragile sites. It is unclear what aspect of telomere structure confers this fragile nature; however, TRF1 is required to prevent these replication problems (Sfeir et al., 2009). Moreover, TRF2 and POT1 function independently to repress DNA damage signaling and DNA repair pathways (Denchi and de Lange, 2007). The specificity of shelterin binding to TTAGGG repeats means that any sequence perturbations in the telomere are likely to have a profound impact on shelterin binding.

**FIGURE 2:**
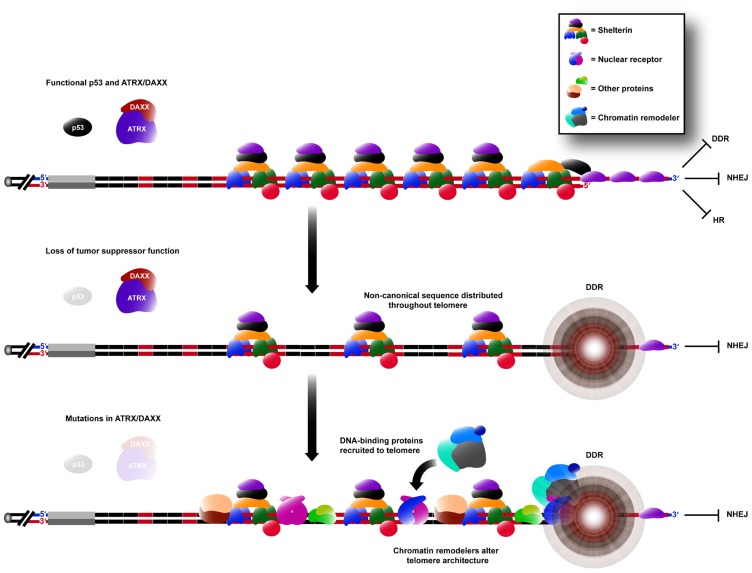
**Remodeling of the telomere architecture during activation of the ALT mechanism**. Non-canonical repeat sequences existing in the proximal region are distributed throughout the telomere array during ALT activation (see **Figure 1**). Hence, there is an insufficient concentration of shelterin binding sites for telomere capping, causing the telomere to elicit a DDR, whilst still being able to suppress chromosomal end-to-end fusions caused by NHEJ. DNA-binding proteins capable of binding specifically to these non-canonical sequences are consequently spread throughout the telomere, increasing its recombinogenicity. These proteins may also be capable of recruiting various chromatin remodeling complexes which can alter the telomere architecture further, in favor of telomeric recombination.

Variant repeat interspersion not only disrupts shelterin binding, but can also be predicted to result in sequence-specific binding of other proteins (**Figure 2**). This is exemplified by the localization of a group of nuclear receptors to the telomeres of ALT cells (Dejardin and Kingston, 2009; Conomos et al., 2012) because of their high binding affinity for the TCAGGG variant repeat (Conomos et al., 2012). It has been demonstrated experimentally that telomeric incorporation of TCAGGG repeats directly resulted in recruitment of nuclear receptors, an increased number of TIFs and the induction of some ALT phenotypic characteristics. It remains to be determined whether other sequences within ALT telomeres are similarly responsible for altered protein binding.

## EPIGENETIC STATE OF ALT TELOMERIC CHROMATIN

It is possible that aspects of telomere architecture other than DNA sequence and shelterin binding also contribute to a state that is permissive for telomeric recombination and ALT activity. Telomeric chromatin carries histone modifications characteristic of transcriptional repression (reviewed in Blasco, 2007; Grewal and Jia, 2007). These include the heterochromatic marks, H3K9me3 and H4K20me3 (trimethylation of histone H3 at lysine 9 and of histone H4 at lysine 20), histone hypoacetylation, and the accumulation of several isoforms of heterochromatin protein 1 (HP1; Garcia-Cao et al., 2004; Benetti et al., 2007a; Michishita et al., 2008). In addition, subtelomeric regions contain hypermethylated DNA (Gonzalo et al., 2006). Repressive chromatin modifications are also enriched at subtelomeric repeats (Benetti et al., 2007a,b), which appears paradoxical given the subtelomeric transcriptional origin of telomeric repeat-containing RNA (TERRA; Azzalin et al., 2007; Schoeftner and Blasco, 2008; Luke and Lingner, 2009). TERRA has also been implicated in the regulation of telomere length and telomeric chromatin structure, having been shown to facilitate heterochromatin formation at telomeres via recruitment of H3K9me3 and HP1 (Deng et al., 2009), and in negative feedback regulation of its own transcription (Arnoult et al., 2012).

The results of several studies predominantly in mice, have suggested that alterations in telomeric chromatin may cause some phenotypic characteristics of ALT, and may ultimately result in ALT activity. Manipulation of mouse telomeric and subtelomeric heterochromatin resulted in a substantially increased number of T-SCEs and telomere elongation (Gonzalo et al., 2006; Benetti et al., 2007a,b). Furthermore, a number of studies have shown that loss of telomeric heterochromatic marks in mice leads to an increase in the number of APBs per cell (Garcia-Cao et al., 2004; Gonzalo et al., 2006; Benetti et al., 2007a,b, 2008). It has been speculated that telomeric chromatin can adopt a more open configuration, thus facilitating HR, ALT-mediated telomere elongation, and APB formation, although increased telomerase activity due to greater access of telomerase to the telomere cannot be excluded as the cause of these alterations. It therefore remains an interesting possibility that a “closed” telomeric and subtelomeric chromatin state is involved in repressing the ALT mechanism (Gonzalo et al., 2006; Benetti et al., 2007a,b).

Decreased subtelomeric DNA methylation, resulting from mutant DNA methyltransferases, was reported to be associated with increased telomeric recombination frequency and telomere lengthening in mice (Gonzalo et al., 2006). Human telomerase-positive cell lines showed a negative correlation of subtelomeric DNA methylation with telomere length and telomere recombination, and treatment of telomerase-positive cell lines with demethylating drugs caused hypomethylation of subtelomeric repeats and increased telomere recombination (Vera et al., 2008). In human ALT cells, however, the relationship between subtelomeric DNA methylation and ALT activity is currently unclear. One study found that the level of subtelomeric DNA methylation was heterogeneous in human ALT cells, but that on average it was similar to the level in the non-immortalized cells from which they were derived, and much less than in telomerase-positive cell lines (Ng et al., 2009). A caveat to this and other studies of subtelomeric DNA methylation is that only a small number of subtelomeric DNA regions at various distances from the telomeres were sampled. It has also been observed that ALT cells have more TERRA than normal cell strains or telomerase-positive cell lines, even when adjusted for the greatly increased telomeric DNA content of ALT cells (Ng et al., 2009). Another study found that there is genome-wide hypomethylation of Alu repeats and pericentromeric Sat2 DNA sequences in ALT-positive human tumor cells, and that although subtelomeric DNA hypomethylation was frequently present in these cells it was not required for HR manifested as T-SCEs (Tilman et al., 2009).

## THE ROLE OF CHROMATIN REMODELING FACTORS IN ALT

Circumstantial evidence for an altered epigenetic state in ALT telomeres was obtained by mass spectrometric analysis of the protein composition of telomeric chromatin (Dejardin and Kingston, 2009). Numerous chromatin remodeling proteins were found to be present at the telomeres of an ALT cell line but were not detected at the telomeres of the telomerase-positive control. Most notably, a class of nuclear receptors, which bind to variant repeats and are capable of initiating gene expression changes via recruitment of chromatin remodelers (Cui et al., 2011), were identified at ALT telomeres. It is possible that recruitment of such proteins may alter the heterochromatic state of ALT telomeres, contributing to the derepression of telomeric recombination.

Recent studies of ALT tumors and immortalized cell lines found a strong correlation between telomere maintenance by ALT and loss of activity of the switch/sucrose non-fermentable (SWI/SNF) family ATP-dependent helicase (ATRX) or its binding partner death-associated protein 6 (DAXX; Heaphy et al., 2011; Bower et al., 2012; Lovejoy et al., 2012; Schwartzentruber et al., 2012). ATRX and DAXX form a chromatin remodeling complex that localizes to PML nuclear bodies (Xue et al., 2003), although the precise mechanism of chromatin remodeling remains elusive. Nevertheless, it has been shown that ATRX and DAXX act in concert to deliver the histone variant H3.3 to telomeres in a replication-independent manner (Goldberg et al., 2010; Law et al., 2010; Lewis et al., 2010). While the purpose of this H3.3 deposition at telomeres is not understood, it has been postulated that inhibition of ATRX/DAXX function may result in the loss of heterochromatic marks thought to suppress the inherently recombinogenic nature of repetitive telomeric DNA.

Some ALT tumors, however, have mutations in both H3.3 and a member of the ATRX/DAXX complex (Schwartzentruber et al., 2012), which indicates that the loss of some function of ATRX/DAXX other than H3.3 deposition is selected for in ALT tumors. ATRX also appears to have a function in the repression of TERRA (Goldberg et al., 2010), which is consistent with the observation that elevated levels of TERRA exist in many ALT tumors and cell lines compared to those which have activated telomerase (Ng et al., 2009; Lovejoy et al., 2012; Sampl et al., 2012). ATRX depletion in mouse embryonic stem cells has also been shown to reduce HP1α recruitment to telomeres and to cause an increase in telomere dysfunction as demonstrated by localization of γ-H2AX at chromosome ends (Wong et al., 2010). Alternatively, loss of ATRX/DAXX function may act elsewhere in the genome and lead to altered gene expression, e.g., by binding to DNA structures such as G-quadruplexes (Law et al., 2010), thus indirectly effecting changes that promote ALT activity. Nonetheless, depletion of either ATRX or DAXX failed to activate ALT in SV40-transformed fibroblasts (Bower et al., 2012; Lovejoy et al., 2012), suggesting that loss of ATRX/DAXX function alone is not sufficient for ALT to be initiated.

## CONCLUDING REMARKS

In light of the evidence reviewed above we propose that remodeling of the telomeric architecture plays a key role in permitting sufficient levels of ALT activity to prevent telomere shortening in ALT cell lines and tumors. Changes in DNA content, in which variant repeat sequences that occur in the proximal region of the telomere become spread throughout the telomeres, are common. This presumably occurs initially via a rare, stochastic event in which the proximal region is used as a copy template by a telomere, but the presence of these sequences in a telomere contributes to a state which is permissive to ALT that results in their spread to other telomeres. Consequences of this altered DNA content include binding of additional proteins as well as a decreased relative shelterin content that may lead to secondary changes in telomeric heterochromatin. Furthermore, other alterations in telomeric chromatin marks may also contribute to the ALT-permissive state, including changes that may result from loss of ATRX/DAXX function, which is a common characteristic of the ALT mechanism.

## Conflict of Interest Statement

The authors declare that the research was conducted in the absence of any commercial or financial relationships that could be construed as a potential conflict of interest.
